# Optimizing Selection and Mating in Genomic Selection with a Look-Ahead Approach: An Operations Research Framework

**DOI:** 10.1534/g3.118.200842

**Published:** 2019-05-20

**Authors:** Saba Moeinizade, Guiping Hu, Lizhi Wang, Patrick S. Schnable

**Affiliations:** * Department of Industrial and Manufacturing Systems Engineering, Iowa State University,; † Department of Agronomy, Iowa State University

**Keywords:** Genetic gain, Genomic, Selection, Look-ahead, Selection, Simulation, Optimization, Genomic Prediction, GenPred, Shared Data Resources

## Abstract

New genotyping technologies have made large amounts of genotypic data available for plant breeders to use in their efforts to accelerate the rate of genetic gain. Genomic selection (GS) techniques allow breeders to use genotypic data to identify and select, for example, plants predicted to exhibit drought tolerance, thereby saving expensive and limited field-testing resources relative to phenotyping all plants within a population. A major limitation of existing GS approaches is the trade-off between short-term genetic gain and long-term potential. Some approaches focus on achieving short-term genetic gain at the cost of reduced genetic diversity necessary for long-term gains. In contrast, others compromise short-term progress to preserve long-term potential without consideration of the time and resources required to achieve it. Our contribution is to define a new “look-ahead” metric for assessing selection decisions, which evaluates the probability of achieving high genetic gains by a specific time with limited resources. Moreover, we propose a heuristic algorithm to identify optimal selection decisions that maximize the look-ahead metric. Simulation results demonstrate that look-ahead selection outperforms other published selection methods.

Feeding the world’s growing population remains a significant challenge. Advances in plant breeding have been instrumental in improving agricultural output. Classical plant breeding programs rely on the phenotyping of progenies in field trials to identify superior individuals. The number of individuals that can be phenotyped is resource limited (Rincent *et al.* 2017), which limits genetic gain. Genomic selection (GS) refers to using a set of markers distributed across the genome to estimate the breeding value of selection candidates for quantitative traits (Goddard 2009). GS makes it possible to predict the performance of unphenotyped individuals from readily available genotyping data (Rincent *et al.* 2017; Meuwissen *et al.* 2001). Genomic Estimated Breeding Value (GEBV) of individual plants (or animals) has been widely adopted as the selection criteria; it selects individuals based on the sum of their estimated marker effects (Meuwissen *et al.* 2001). This approach has been widely adopted in GS practice due to its effectiveness in achieving short-term genetic improvements. More recently, two methods have been proposed to improve conventional GS (CGS): the optimal haploid value (OHV) (Daetwyler *et al.* 2015) and the optimal population value (OPV) (Goiffon *et al.* 2017). Simulation experiments and some empirical studies have shown that CGS selection results in rapid genetic gains (Hayes *et al.* 2009; Lorenzana and Bernardo 2009; VanRaden *et al.* 2009; Jannink 2010). However, CGS focuses on one or two cycles of selection and does not guarantee long-term gain (Sonesson *et al.* 2012; Lin *et al.* 2017; Gorjanc *et al.* 2018; Akdemir *et al.* 2019). The OHV method, calculates the GEBV of the best possible doubled haploid (DH) derived from an individual (Daetwyler *et al.* 2015). This method focuses selection on haplotypes and optimizes the breeding program toward the end goal of generating an elite fixed line (Daetwyler *et al.* 2015). Simulation studies have shown that OHV selection results in more genetic gain and diversity as compared to CGS (Daetwyler *et al.* 2015). CGS and OHV are truncation selection approaches in that they rank *individuals* and select the top fraction of the population. In contrast, OPV is a group-based selection strategy. Specifically, OPV selects the best *group* of individuals based on their interactive effects and calculates the GEBV of the best possible progeny from this group produced after an unlimited number of generations, which may require a large amount of time and resources to achieve (Goiffon *et al.* 2017). In this paper, we extend OPV by again selecting groups of individuals as a unit, but propose an innovative method for selecting groups, “look-ahead selection” (LAS). This new selection method can improve genetic gain by maximizing the expected GEBV of the best offspring in the terminal generation. It makes the optimal trade-off between short-term gain and long-term potential to achieve the highest genetic gain within a specified time.

## Materials and Methods

### Generic formulation for GS methods

In this section, we present a generic formulation for existing GS methods namely, CGS, OHV, OPV, and the new selection method, LAS. Equations (1), (2), and (3) show this genetic optimization formulation.

$$\begin{array}{cc} {\text{max}}_{x} & f\left(x\right) \end{array}$$ (1)

$$\begin{array}{cc} \text{such}\;\text{that} & \sum_{n=1}^{N}x_{n}=S \end{array}$$ (2)

$$x_{n}\in \left\{0,1\right\},n\in \left\{1,\ldots ,N\right\}$$ (3)

Here,

- *N* is the number of individuals in the population.
- $x_{n}$ is a binary decision variable that shows whether individual *n* is selected ($x_{n}=1$) or not ($x_{n}=0$).
- *S* is the number of individuals that are to be selected out of the current population.

It should be observed that the only difference among the three previous methods is in their objective functions as they aim to maximize different objectives. The objective function of the optimization problem, f(x) is formulated as $f{\left(x\right)}^{CGS}$, $f{\left(x\right)}^{OHV}$, and $f{\left(x\right)}^{OPV}$ in equations (4), (5), and (6) respectively.

#### Conventional genomic selection:

Meuwissen *et al.* (2001) proposed to evaluate an individual as a breeding parent by its genomic estimated breeding value (GEBV), which is the sum of all marker effects across the entire genome, as defined in equation (4). The CGS method selects individuals with the highest GEBVs.

$$f{\left(x\right)}^{\text{CGS}}=\sum_{n=1}^{N}\sum_{l=1}^{L}\sum_{m=1}^{2}G_{l,m,n}\beta_{l}x_{n}.$$ (4)

Here, the notations are defined as follow:

- *L*: The number of marker loci.
- $G_{l,m,n}\in \left\{0,1\right\},\forall l\in \left\{1,2,\ldots ,L\right\},\forall m\in \left\{1,2\right\}\;\text{and}\;\forall n\in \left\{1,2,\ldots ,N\right\}$: The genotypic information of locus *l* from chromosome *m* of individual *n*, with 1 and 0 representing the major and minor allele, respectively.
- $\beta_{l}$: The normalized effect of the major allele at locus *l*, with that for the minor allele being 0.
- *M*: The ploidy of the plants. We use diploid species (*M* = 2) as an example in this paper.

To maximize long-term response, the weighted genomic selection (Goddard 2009; Jannink 2010) was proposed as a variation of the CGS method by emphasizing the preservation of rare favorable alleles. It replaced the allele effect $\beta_{l}$ in equation (4) with $\frac{\beta_{l}}{\sqrt{\text{max}\left(w_{l},1/N\right)}}$, where $w_{l}$ is the frequency of favorable alleles at locus *l* among all individuals in the population. As such, this variation gives a higher weight to low-frequency favorable alleles. Notice that the denominator $\sqrt{\text{max}\left(w_{l},1/N\right)}$ is equal to $\sqrt{w_{l}}$ except for $w_{l}=0$ when $G_{l,m,n}=0$ for all *m* and *n*.

#### Optimal haploid value:

More than a decade after the CGS metohd, OHV was proposed to combine the creation of doubled haploids with GS methods and evaluates the potential of producing elite doubled haploids (Daetwyler *et al.* 2015). Equation (5) shows the objective function for OHV selection. This method selects individuals with the highest OHVs.

$$f{\left(x\right)}^{\text{OHV}}=2\sum_{n=1}^{N}\sum_{b=1}^{B}\underset{m\in \left\{1,2\right\}}{\text{max}}\sum_{l\in H\left(b\right)}G_{l,m,n}\beta_{l}x_{n}.$$ (5)

Here, segments of adjacent markers are clustered into haplotypes, which are defined as follows:

- *B*: The number of haplotype blocks per chromosome.
- H(b),∀b∈{1,…,B}: The set of marker loci that belong to haplotype block *b*.

The OHV of an individual is the GEBV of its best possible DH progeny. Recombination events are assumed to be possible between haplotypes but not within them. This assumption reduces the computational effort of the algorithm.

What also makes CGS and OHV computationally efficient is the fact that they are both truncation selection methods, which assumes that the contribution of breeding parents are separable and additive. Mathematically, the summation operator ${\sum^{\text{​}}}_{n=1}^{N}$ in equation (4) and (5) suggests that the maximization of the objective functions $f{\left(x\right)}^{\text{CGS}}$ or $f{\left(x\right)}^{\text{OHV}}$ can be easily achieved by evaluating each individual *n* separately and setting $x_{n}=1$ for the ones with the highest GEBVs or OHVs. Compared with CGS, OHV represents an important shift of the selection objective from maximizing genetic achievement of the parents to that of their progeny.

#### Optimal population value:

OPV selection is an extension to OHV which evaluates the breeding merit of a set of individuals instead of evaluating the breeding value of a single individual (Goiffon *et al.* 2017). The OPV of breeding population *S* is the GEBV of the best possible progeny produced after an unlimited number of generations. The objective function for the OPV method is defined as follows:

$$f{\left(x\right)}^{\text{OPV}}=2\sum_{b=1}^{B}\underset{n\in \left\{1,\ldots ,N\right\}}{\text{max}}\underset{m\in \left\{1,2\right\}}{\text{max}}\sum_{l\in H\left(b\right)}G_{l,m,n}\beta_{l}x_{n}.$$ (6)

OPV represents another important shift of the selection objective from individual-based truncation selection to group-based selection. The contribution of a breeding parent is evaluated based on not only the favorable alleles that it carries but also the favorable alleles that it carries but are missing in other selected breeding parents. A limitation of OPV is that the objective function $f{\left(x\right)}^{\text{OPV}}$ is a lot harder to optimize, since it is no longer separable with respect to *x*. As a result, heuristic algorithms were used to identify good but not necessarily optimal selections.

### Potential improvements

The success of CGS has been demonstrated in numerous simulation and field experiments, especially in achieving short-term genetic gains in both plant and animal breeding (Meuwissen 1997; Rosvall 1999; Hayes *et al.* 2002; Ullrich 2007; Hayes *et al.* 2009; Lorenzana and Bernardo 2009; VanRaden *et al.* 2009; Jannink *et al.* 2010; Mujibi *et al.* 2011; Nakaya and Isobe 2012; Hallatschek and Geyrhofer 2015). OHV and OPV were proposed as extensions of CGS to improve long-term genetic gains, which have been shown to be effective in simulation studies. Herein, we identify three areas in genomic selection that can be made more efficient and present a new genomic selection method that attempts to address each of these three areas.

First, time management. For a given population of individuals, the optimal selection decision should depend on whether the deadline of the breeding project is in the near future or far down the road. However, none of the aforementioned three methods take deadlines into consideration.

Second, mating strategy. All three methods focus on selecting breeding parents without explicitly indicating how they should be mated in pairs, but several studies have observed that different mating decisions may affect genetic gain (Toro and Varona 2010; Kinghorn 2011; Sun *et al.* 2013; Akdemir and Sánchez 2016; Liu *et al.* 2017; Wang *et al.* 2018).

Third, resource allocation. Intuitively, making more crosses and producing more progenies leads to a higher chance of creating outstanding individuals from the progeny population, but this also requires more resources. Allocating a fixed total budget over a period of time to achieve the best final outcome is therefore a strategic decision that needs to be optimized (Lorenz 2013).

### Look-ahead selection

The cornerstone of the LAS method is a new definition of the objective function, $f^{\text{LAS}}\left(x,y,r,T-t\right)$, that reflects what truly matters in genomic selection. The input of this function includes selected breeding parents (*x*), mating decisions (*y*), recombination frequencies (*r*), and remaining number of generations (T−t, the difference between the current generation number *t* and the deadline *T*). The former two input terms are decision variables that need to be optimized by the model, whereas the latter two are parameters that the model needs to take into account when searching for the optimal solution. We define $f^{\text{LAS}}$ as the *expected GEBV of the best offspring in the terminal generation*. In comparison, $f^{\text{CGS}}$ can be interpreted as the genetic achievement of the breeding parents measured in terms of GEBV; and $f^{\text{OHV}}$ and $f^{\text{OPV}}$ represent the best possible progeny that can be produced by, respectively, self pollination and cross pollination, both assuming unlimited time and resources. The models for these three methods only differ in the objective functions but share the same constraints (2) and (3), whereas the LAS model requires additional constraints. The LAS method can be formulated as follows.

$$\begin{array}{cc} \underset{x,y}{\text{max}} & f^{\text{LAS}}\left(x,y,r,T-t\right) \end{array}$$ (7)

$$\begin{array}{c} \text{such}\;\text{that}\;\text{Constraints}\;\left(\text{2}\right)\;\text{and}\;\left(\text{3}\right) \end{array}$$ (8)

$$\begin{array}{ll} x_{n}=\sum_{j=1}^{N}y_{n,j} & \forall n\in \left\{1,\ldots ,N\right\} \end{array}$$ (9)

$$\begin{array}{cc} y_{i,j}\in \left\{0,1\right\} & \forall i,j\in \left\{1,\ldots ,N\right\} \end{array}$$ (10)

The new variables and parameters are defined as follows.

- $y_{i,j}$: A binary variable that shows whether individual *i* is mated with individual *j* ($y_{i,j}=1$) or not ($y_{i,j}=0$).
- $r\in {\left[0,0.5\right]}^{L-1}$: The recombination frequency vector.

The remainder of this section will explain how to numerically evaluate the objective function $f^{\text{LAS}}\left(x,y,r,T-t\right)$ for any given solution (x,y), how to search for the optimal (or close to optimal) solution $\left(x^{\text{*}},y^{\text{*}}\right)$ that achieves the maximal value in the objective function, and how to allocate resources to improve the rate of genetic gains.

#### Evaluation of the objective function $f^{\mathrm{LAS}}$:

**Figure 1:**
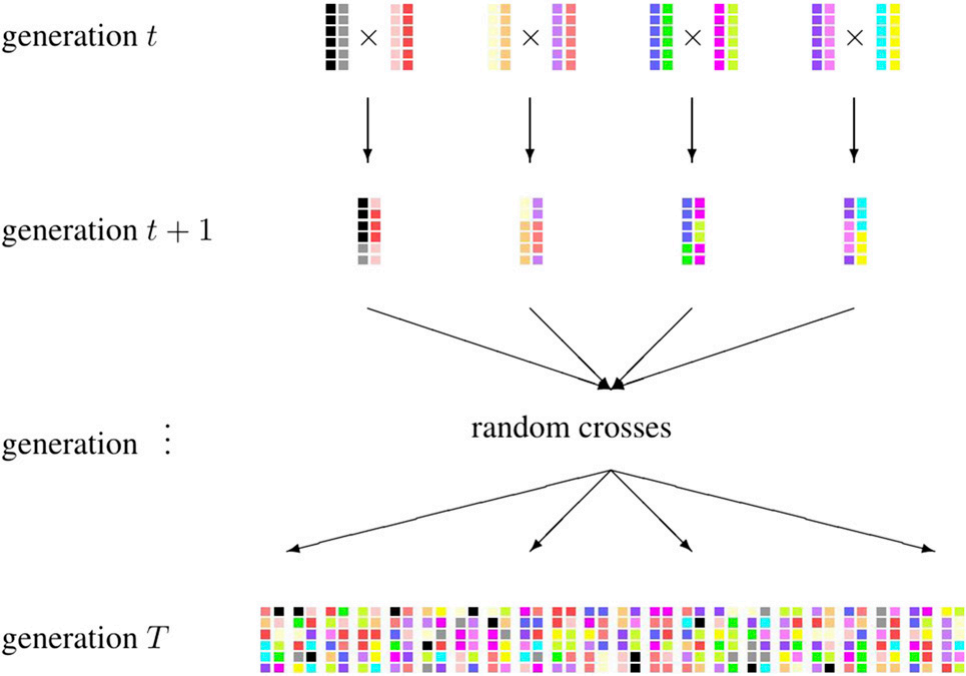
The look-ahead simulation.

The exact evaluation of the objective function $f^{\text{LAS}}$ is challenging both computationally and analytically due to uncertain recombination events over T−t generations as well as the selection, mating, and resource allocation decisions that will be made therein. To overcome this challenge, we designed a novel simulation method that provides a computationally tractable yet reasonable approximation of the true objective function. Figure 1 illustrates the look-ahead simulation that is based on two simplifying assumptions.

**Assumption 1:** The selected pairs of breeding parents will each produce one progeny in generation t+1.
**Assumption 2:** All progenies from generation t+1 to T−1 were crossed with each other (including selfing) in the same generation, each producing one progeny.

As such, the objective function $f^{\text{LAS}}$ can be approximated by taking a random sample of the population in generation *T* of the look-ahead simulation and calculating the highest GEBV of all individuals.

The following theorem defines the distribution of the progenies in the final generation *T*, which allows efficient evaluation of the approximated objective function.

**Theorem 1.** Let $G\in {\left\{0,1\right\}}^{L\times 2\times S}$ denote the genotype of a population in generation t with an even number, *S*, of individuals. Suppose all individuals with odd indices, {1,3,…,S−1}, are respectively mated with the next individuals, {2,4,…,S}. These individuals are mated according to Assumptions 1 and 2. Let $g\in {\left\{0,1\right\}}^{L}$ denote a random gamete produced by breeding parents in meiosis of the (T−1)st generation. The distribution of *g* can be described by the following equations (11) and (12)
  $$P\left(g_{1}=G_{1,m,i}\right)=\frac{1}{2S},\forall i\in \left\{1,2,\ldots ,S\right\},\forall m\in \left\{1,2\right\}.$$ (11)
  $$P\left(g_{l+1}=G_{l+1,m^{1},i^{1}}|g_{l}=G_{l,m^{0},i^{0}}\right)$$ (12)
  $$=\{\begin{array}{cc} {\left(1-r_{l+1}\right)}^{2}\left(1-R_{l+1}\right), & \text{if}\;\;i^{0}=i^{1}\;\text{and}\;m^{0}=m^{1}\\ r_{l+1}\left(1-r_{l+1}\right)\left(1-R_{l+1}\right), & \text{if}\;\;i^{0}=i^{1}\;\text{and}\;m^{0}\neq m^{1}\\ \frac{1}{2}\;r_{l+1}\left(1-R_{l+1}\right), & \text{if}\;\;\left\lceil \frac{i^{0}}{2}\right\rceil =\left\lceil \frac{i^{1}}{2}\right\rceil \\ \frac{R_{l+1}}{2\left(S-2\right)}, & \text{if}\;\;\left\lceil \frac{i^{0}}{2}\right\rceil \neq \left\lceil \frac{i^{1}}{2}\right\rceil \end{array},$$
  $$\forall l\in \left\{1,\ldots ,L-1\right\},\;\forall i^{0},i^{1}\in \left\{1,2,\ldots ,S\right\},\;\forall m^{0},m^{1}\in \left\{1,2\right\}.$$

Here, $r\in {\left[0,0.5\right]}^{L-1}$ is the given vector of recombination frequencies and $R_{l}$ is the recombination frequency between allele l and allele l+1 between generations t+2 and *T* for all l∈{1,…,L−1}, which can be derived as:

$$R_{l}=\frac{\left(S-2\right)\left[1-{\left(1-r_{l}\right)}^{T-t}\right]}{S}.$$ (13)

The proof for equation (13) is provided in the appendix.

#### Optimization of the objective function $f^{\mathrm{LAS}}$:

Unlike truncation selection methods CGS and OHV, which are easy to optimize due to separable objective functions with respect to the selection decision *x*, the OPV and LAS methods require the optimization of the selected breeding parents’ synergistic contribution. A heuristic algorithm was designed to optimize $f^{\text{OPV}}$ in Goiffon *et al.* (2017), where a randomly selected set of breeding parents is iteratively updated to maximize the $f^{\text{OPV}}$ function through pairwise swaps between a selected individual and every other unselected one. A similar heuristic can also be applied to optimize the $f^{\text{LAS}}$ function with two minor points of caution. First, OPV only selects individuals, while in contrast, LAS also pairs them up, so the orders of the selected individuals in generation *t* must be preserved to reflect the mating strategy. Second, constraint (2) ensures fair comparison between the four methods by specifying the number of selected individuals. This constraint helps CGS and OHV by maintaining genetic diversity. On the other hand, maintaining genetic diversity is a built-in feature in OPV and LAS methods. Hence, the decision maker can choose to relax constraint (2) on OPV or LAS methods in cases that selfing or polygamous crosses are beneficial.

#### Heuristic strategy for resource allocation:

There are two dimensions of resource allocation in genomic selection (beyond genomic prediction of allele effects): allocation of total budget across a number of generations and allocation of the given budget for a specific generation over multiple crosses. In this paper, we assumed equal temporal allocation of the total budget over the breeding duration and hence a fixed number of crosses and population size for each generation. The proposed heuristic strategy attempted to accelerate the rate of genetic gain by strategically varying the numbers of progenies produced from different crosses based on the genetic diversity of the breeding parents. Let $n_{1}$ and $n_{2}$ be the indices of the two breeding parents (that have been selected and paired according to the LAS method) in the current generation with *G* representing its genotype, then the genetic diversity is defined as

$$\sum_{l}\left(\underset{\begin{array}{c} n\in \left\{n_{1},n_{2}\right\}\\ m\in \left\{1,2\right\} \end{array}}{\text{max}}G_{l,m,n}\beta_{l}-\underset{\begin{array}{c} n\in \left\{n_{1},n_{2}\right\}\\ m\in \left\{1,2\right\} \end{array}}{\text{min}}G_{l,m,n}\beta_{l}\right),$$ (14)

which is the aggregated range of GEBVs over all haplotype blocks. Given a fixed budget for the current generation, the numbers of progenies produced from multiple crosses are set to be proportional to the genetic diversity measures of the breeding parents. The rationale for this heuristic is to spend more resources on those crosses that have wider predicted phenotypic distributions and thus higher probabilities of producing outstanding progenies.

### Data availability

All data including phased single nucleotide polymorphisms (SNPs) for maize inbred lines from the Shoot Apical Meristem (SAM) Diversity Panel and genetic maps are available at Figshare: https://iastate.figshare.com/s/374176500b04fd6f3729.

## Results

### Simulation setting

In this paper, the genotypic data ($G_{l,m,n}$), marker effects ($\beta_{l}$) and recombination rates ($r_{l}$) are based on Goiffon *et al.* (2017). The genotypic data contains genotypes of 369 maize inbred lines consisting of L=1,406,757 SNPs distributed across ten maize chromosomes. Marker effects were estimated on the basis of 369 shoot apical meristem phenotypes (Leiboff *et al.* 2015) using the BayesB model (Meuwissen *et al.* 2001). Similar to Goiffon *et al.* (2017), we assumed that marker effects were known and that errors in marker effects have an equal effect on all selection methods. The genetic map developed from maize nested association mapping (NAM) population is used for estimating recombination rates (Yu *et al.* 2008). To facilitate comparisons, genetic data were scaled such that the maximum potential of the initial breeding population is 100.

**Figure 2:**
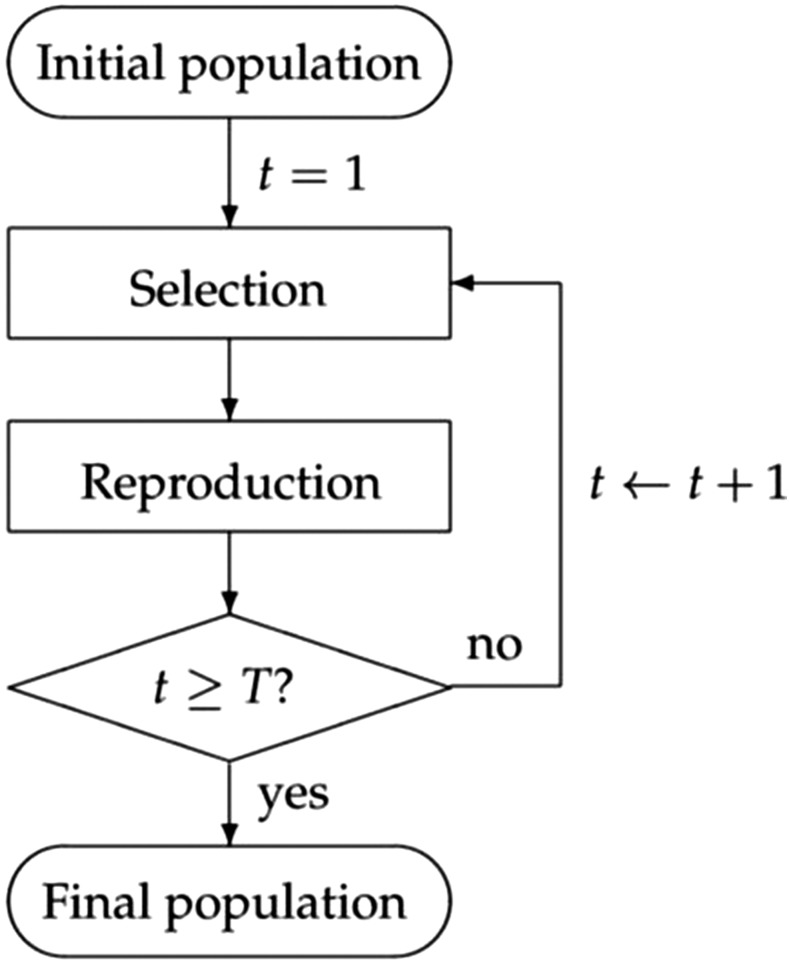
The simulation diagram, adopted from Goiffon *et al.* (2017).

The same simulation process (shown in Figure 2) as (Goiffon *et al.* 2017) was used to compare the four methods in our study. Each of the components in Figure 2 is explained as follows:

- **The *initial population* start point**: In plant breeding, the genomic selection process starts with an initial population of individuals. The genotypes and marker effects are given at this point. In each simulation run, 200 individuals were selected randomly from the 369 maize inbred lines to make the initial population. Furthermore, the same set of 200 individuals were used as the initial population for all methods to make comparisons consistent.
- **The *selection* step:** All four methods were used to make selection decisions in this step, including mating strategies, number of crosses to make (nc) and number of progenies per cross (np). In particular,
  For **CGS**: S=20 individuals with the highest GEBVs were selected and randomly mated to make nc=10 crosses, each producing np=20 progenies, maintaining a constant population size of 200.
  For **OHV**: S=20 individuals with the highest OHVs were selected and randomly mated to make nc=10 crosses, each producing np=20 progenies, maintaining a constant population size of 200. The same values of B=12 and F=70% as Goiffon *et al.* (2017) were used in our simulation where *F* is the percentage of individuals with the lowest GEBVs removed before optimizing the selected population.
  For **OPV**: S=20 individuals with the highest OPVs were selected and randomly mated to make nc=10 crosses, each producing np=20 progenies, maintaining a constant population size of 200. The same values of B=1 and F=40% as Goiffon *et al.* (2017) were used in our simulation.
  For **LAS**: S=20 individuals were selected and mated according to the look-ahead algorithm to make nc=10 crosses. The number of progenies for each cross was determined by the heuristic strategy described in Section 2.3.3 with the constraint that the total number of progenies remains 200.
- **The *reproduction* step:** The selected individuals were crossed to make the breeding population for the next generation. A random progeny inherits the genetic information from its breeding parents according to *inheritance distribution* defined in Han *et al.* (2017). Let $P\in {\left\{0,1\right\}}^{L\times 2}$ denote the genotype of a random progeny produced from crossing individuals $n_{1}$ and $n_{2}$. Then *P* is determined as follow:
  $$P_{i,j}=G_{i,J_{i}^{j}+1,n_{j}},\forall i\in \left\{1,\ldots ,L\right\},j\in \left\{1,2\right\},$$

where

$$J_{1}=\{\begin{array}{lll} 0, & \text{w}.\text{p}. & 0.5\\ 1, & \text{w}.\text{p}. & 0.5 \end{array},$$ (15)

$$J_{i}=\begin{array}{lll} J_{i-1} & \text{w.p}. & 1-r_{i-1}\\ 1-J_{i-1} & \text{w.p}. & r_{i-1} \end{array},\forall i\in \left\{2,\ldots ,L\right\}.$$ (16)

Here, “w.p.” stands for “with probability”.

- **The** t>=T? **condition:** The breeding cycle repeats itself until generation *T*, a predetermined deadline.
- **The *final population* end point:** After the terminal generation, the population will be assessed to determine its genetic improvement over the initial population.

### Simulation results

One thousand independent simulation repetitions were performed for each of the four selection approaches. Simulations were conducted on a computer with 256GB RAM and a processor with the following specifications: Intel(R) Xeon(R) CPU E5-4650 0 @2.70GHz 2.70GHz (2 processors). The computation time required for one simulation (including 4 methods) was 6248 sec. Hence, it takes almost 1735 Hours (72 days) to conduct 1000 simulations. Ten different simulations have ran in parallel to reduce the CPU calender time to 7 days. The LAS method is modestly more computationally intensive. LAS requires approximately two times more computational time than the other three methods. Major results are summarized as follows.

#### Genetic gains over ten generations:

**Figure 3:**
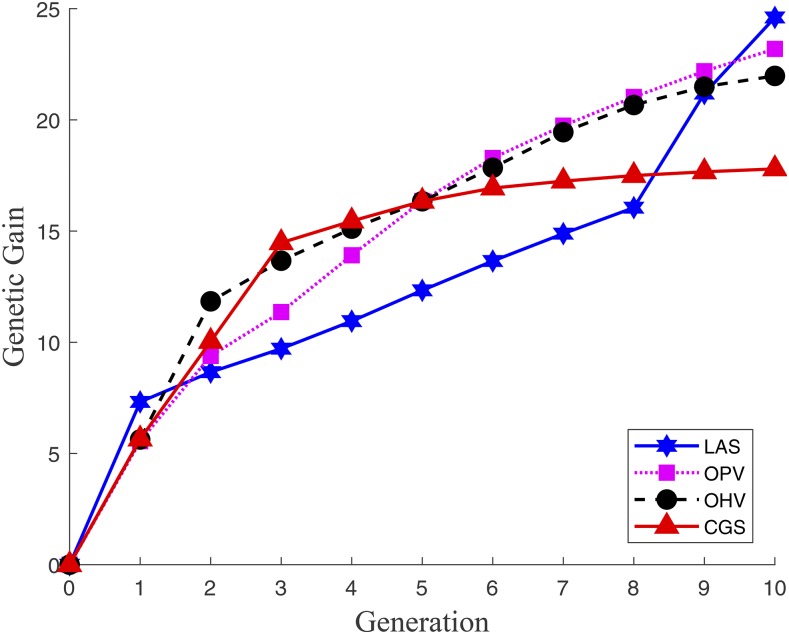
Cumulative genetic gains over 10 generations for four GS methods.

Figure 3 shows the average cumulative genetic gains over ten generations. We define the cumulative genetic gain as the difference between the mean GEBV of the current population and that of the initial population. Because this figure shows genetic gains for each of the four methods averaged across 1,000 simulation repetitions, the comparison reflects their different performances in general. CGS achieved a high rate of genetic gain in the first three generations before gradually reaching a plateau. OHV maintained a relatively high rate of genetic gain throughout ten generations due to its emphasis on the progenies rather than the parents. OPV managed to achieve an even higher genetic gain by the terminal generation at the cost of lower rate of genetic gains in early generations, which is attribute to its group-based selection strategy that aims to achieve long-term genetic gains by combining desirable alleles from multiple breeding parents. LAS demonstrated a deadline-conscious strategy that patiently stays as an underdog in early generations while accumulating desirable alleles but ultimately surpasses all other methods in the final generation. These results suggested that LAS is capable of making a trade-off between achieving short-term genetic gain and preserving long-term growth potential.

#### Genetic diversity over ten generations:

**Figure 4:**
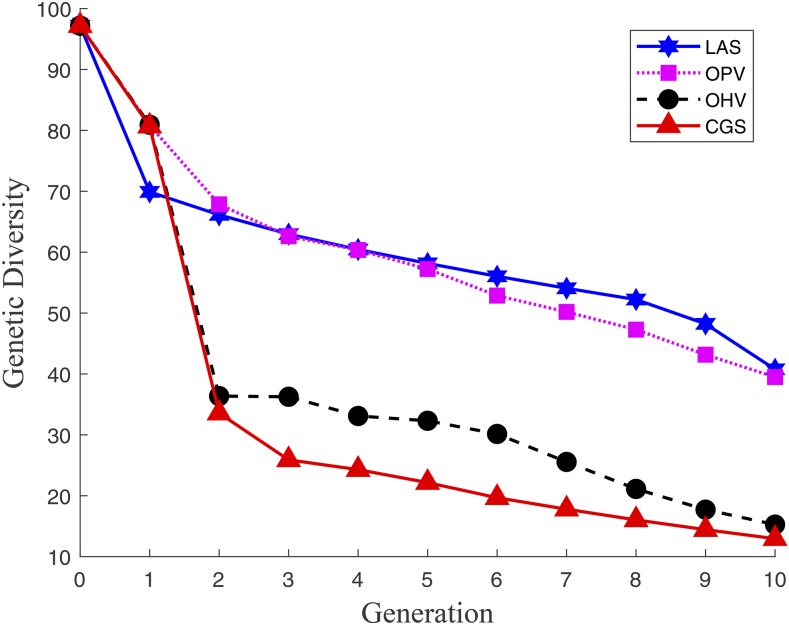
Genetic diversity over 10 generations for four GS methods.

Figure 4 displays the average genetic diversity (defined in equation (14)) over ten generations. The genetic diversity of the two truncation selection methods, CGS and OHV, dropped to about 35% of its initial value in the first two generations, which further deteriorated to about 15% in generation ten. In contrast, the two group-based selection methods, OPV and LAS, maintained genetic diversity at about 65% and 40% in generations two and ten, respectively. These results demonstrated the advantages of group-based selection methods over truncation-based methods in terms of preserving long-term genetic diversity.

#### Genetic gains with varying deadlines:

**Figure 5:**
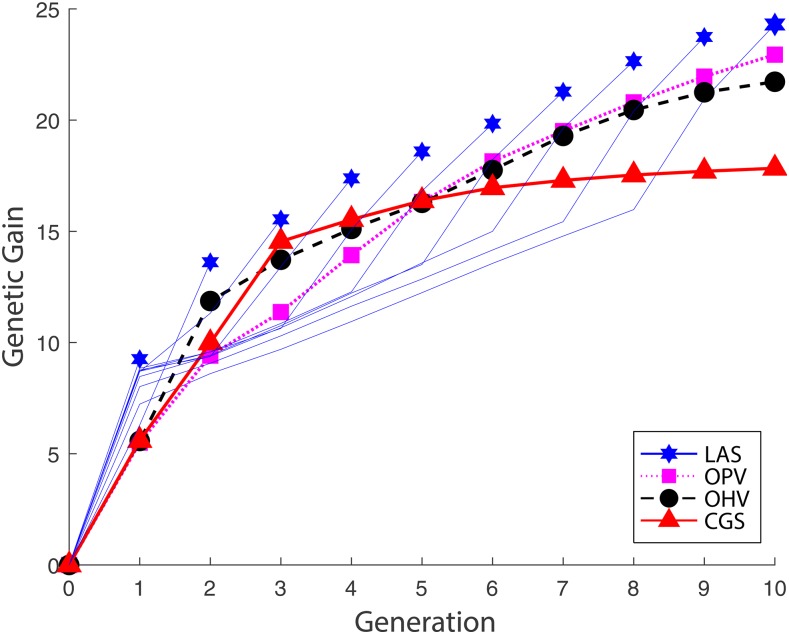
Genetic gains with varying deadlines from T=1 to T=10. LAS adjusts selection decisions based on the user-defined deadline whereas other three methods always make the same selection decisions.

LAS is the only method that adjusts selection decisions based on the user-defined deadline. Figure 5 shows the performance of LAS with varying deadlines from T=1 to T=10. In all ten cases, LAS used a similar strategy to patiently accumulate desirable alleles in early generations and make big leaps in the final two generations. As a result, LAS outperformed all other methods for all tested deadlines. The other three methods make the same selection decisions and thus result in the same performance under different deadlines.

#### Variable performance across different simulation repetitions:

The average values and standard deviations (among the 1,000 simulation repetitions) for population minimum, mean, and maximum in the 10^th^ generation are summarized in Table 1.

**Table 1:** Average values and standard deviations (among the 1,000 simulation repetitions) for population minimum, mean, and maximum in the 10^th^ generation for four selection methods

Method	Min	Mean	Max
CGS	54.88±3.20	55.06±3.23	55.24±3.26
OHV	58.31±4.27	58.95±3.87	59.48±3.84
OPV	57.56±3.73	60.17±3.97	62.16±4.68
LAS	56.58±3.97	61.53±3.83	64.69±4.25

**Figure 6:**
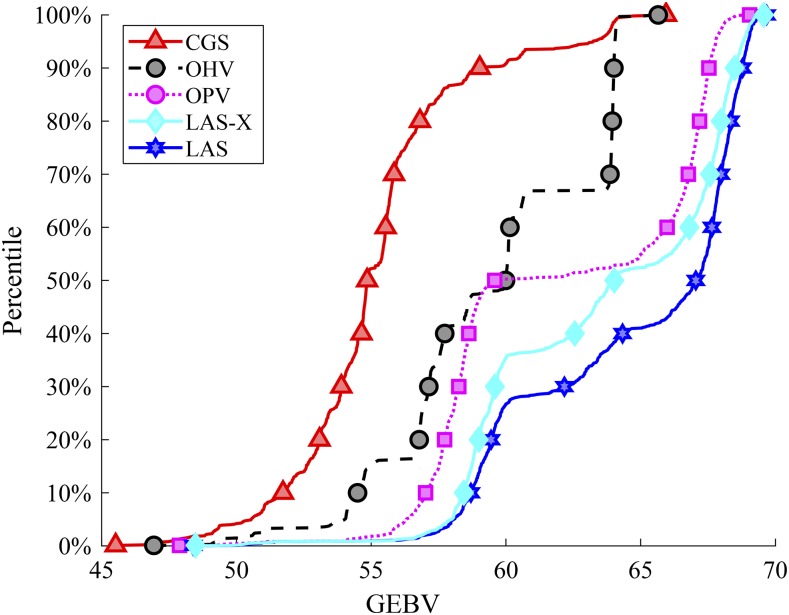
CDFs of population maximum, here LAS-X is the modified LAS method without resource allocation.

Figure 6 compares the cumulative distribution functions (CDFs) of the population maximum in generation 10. Here, the horizontal axis shows the GEBV of an individual (representing genetic gains) whereas the vertical axis is the percentile of the simulation repetitions. By definition, the 1^st^ percentile is one of the worst performances within the 1,000 simulation repetitions, the 99^th^ percentile is one of the best, and the 50^th^ percentile is the median value. As such, the further toward the right and bottom directions of the figure a CDF curves, the better performance a method has. The figure shows the improvements of different methods from CGS to LAS. In particular, LAS-X is a reduced version of LAS using the same resource allocation strategy with all previous methods (producing the same number of progenies from each cross), rather than using the heuristic strategy for resource allocation described in Section 2.3.3. These results demonstrated the effectiveness of LAS in making selection, mating, and resource allocation decisions.

#### Behavior of LAS in the final two generations:

LAS has an interesting behavior in the final two generations when it makes big leaps in genetic gain (Figures 3 and 5). This happens because LAS accumulates desirable alleles in the early generations to utilize in the final generations.

**Figure 7:**
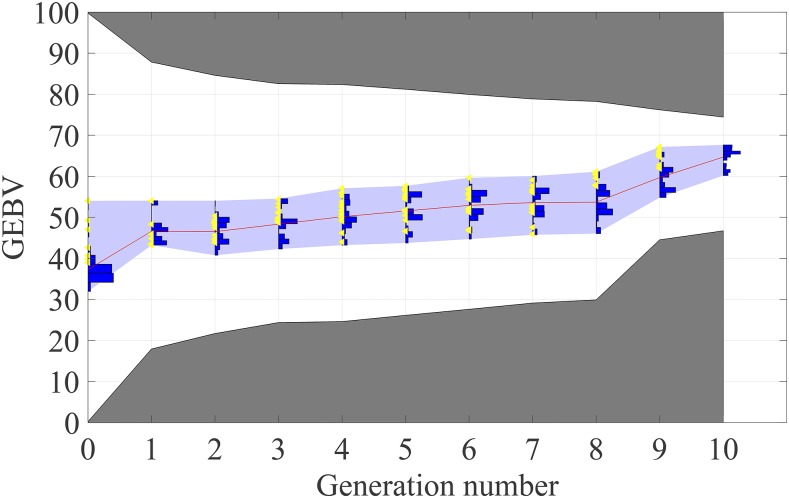
A sample simulation result using the LAS method presenting histograms of population GEBVs over time. Here, the red curve is the mean of population GEBVs and the boundaries of white and gray areas are the upper and lower selection limits. For a given generation, the upper selection limit shows the maximum potential of population in terms of GEBV values and similarly the lower selection limit shows the minimum potential of the population. The maximum, mean and minimum GEBVs are respectively 67.64, 64.69, and 60.18 in the final generation.

Figure 7 presents histograms of population GEBVs over time for one sample simulation using the LAS method. The yellow triangles show the GEBV of selected breeding parents from the population in each generation. This demonstrates how the breeding value rankings of the individuals selected by LAS change by generation. Note that in the last two generations LAS selects individuals with high GEBVs. This explains the behavior of LAS in the final two generations.

### CONCLUSIONS

Genomic selection has been instrumental in improving the efficiency of plant breeding. In this study, we introduced a new selection method, LAS, which has the potential to further improve the efficiency of breeding given limited resources and specific user-defined project duration.

Unlike previous methods which try to maximize the genetic achievement of breeding parents or the best possible progeny without considering time and resource constraints, LAS is maximizing what exactly matters in a GS problem by aiming at the right objective. The objective of LAS is to maximize the expected GEBV of the best offspring in the terminal generation given a limited amount of resources. As such, this method is much more computationally challenging than previous ones, due to multiple complex factors such as recombination frequencies, mating strategy, time management, and resource allocation that are explicitly accounted for. To deal with these challenges, we designed a simulation optimization algorithm that estimates and maximizes the LAS objective function by exploring the selection and mating solution space efficiently.

LAS makes three major contributions to the literature on genomic selection. First, LAS is deadline sensitive. Selection decisions adjust to the project duration to make a trade-off between achieving short-term genetic gains and maintaining genetic diversity long-term. Second, LAS optimizes both selection and mating strategies. It recognizes the importance of mating strategies and assigns selected individuals into pairs of breeding parents to achieve further genetic gains. Third, LAS involves resource allocation decisions. Rather than producing the same number of progenies from each cross, it allows breeding parents with higher genetic diversity to produce more progenies to increase the chance of producing high performers.

LAS was compared with previous genomic selection methods in a comprehensive simulation study using empirical data from a population of inbred maize lines. Computational results demonstrated the improvements of LAS over other methods in three perspectives: (1) LAS achieved the highest genetic gain by the deadline of the breeding project, which varied from one generation to ten generations. (2) LAS preserved the highest level of genetic diversity at the end of the breeding project. (3) LAS outperformed all other methods in almost all percentiles in the 1,000 simulation repetitions.

Future research is needed to address the limitations of the LAS method. The first assumption described in Section 2.3.1 is allowing only one progeny to be produced from the selected pairs of breeding parents in generation t+1 and the second assumption is allowing the crosses to be made within the same generation each producing one progeny from generation t+1 to T−1. These two assumptions were made to simplify the computational requirement of estimating the objective function, which inevitably reduced its accuracy. Moreover, future studies can explore more comprehensive comparisons by performing simulations by: 1. using other methods for estimating marker effects such as GBLUP and ridge regression; 2. considering populations with different LD structures; and 3. applying different resource allocation strategies.

## APPENDIX

### PROOF FOR THEOREM 1

This appendix proves theorem 1 through an example to provide a more insightful description for four different possibilities of recombination. Let’s assume we start with three pairs of breeding parents (S=6). We represent the genotypic information of these individuals with the following matrices:

$$\text{pair one}:\left[\begin{array}{cc} G_{1,1,1} & G_{1,2,1}\\ G_{2,1,1} & G_{2,2,1}\\ \vdots & \vdots \\ G_{L,1,1} & G_{L,2,1} \end{array}\right]\times \left[\begin{array}{cc} G_{1,1,2} & G_{1,2,2}\\ G_{2,1,2} & G_{2,2,2}\\ \vdots & \vdots \\ G_{L,1,2} & G_{L,2,2} \end{array}\right]$$

$$\text{pair two}:\left[\begin{array}{cc} G_{1,1,3} & G_{1,2,3}\\ G_{2,1,3} & G_{2,2,3}\\ \vdots & \vdots \\ G_{L,1,3} & G_{L,2,3} \end{array}\right]\times \left[\begin{array}{cc} G_{1,1,4} & G_{1,2,4}\\ G_{2,1,4} & G_{2,2,4}\\ \vdots & \vdots \\ G_{L,1,4} & G_{L,2,4} \end{array}\right]$$

$$\text{pair three}:\left[\begin{array}{cc} G_{1,1,5} & G_{1,2,5}\\ G_{2,1,5} & G_{2,2,5}\\ \vdots & \vdots \\ G_{L,1,5} & G_{L,2,5} \end{array}\right]\times \left[\begin{array}{cc} G_{1,1,6} & G_{1,2,6}\\ G_{2,1,6} & G_{2,2,6}\\ \vdots & \vdots \\ G_{L,1,6} & G_{L,2,6} \end{array}\right]$$

The individuals in each pair are crossed to produce one progeny. The resulting progenies are then randomly mated for T−t−1 generations. $g\in {\left\{0,1\right\}}^{L}$ is the random gamete produced by breeding parents in meiosis of the (T−1)st generation. From equation (12) we see that four possibilities exist for recombination. Here, we illustrate those four cases with color coding.

*Proof:* We divide the process into two phases: *Phase 1*: generation 0 until 2 and *Phase 2*: generation 2 until *T*. Let $h\in {\left\{0,1\right\}}^{L}$ denote the genotype of a specific gamete produced in meiosis by a progeny of a specific pair of breeding parents from the breeding population. This specific gamete contains the allele $G_{l,m^{0},i^{0}}$ that is passed on to the gamete *g* at locus *l*, *i.e.*, $h_{l}=g_{l}=G_{l,m^{0},i^{0}}$. We know that such a gamete uniquely exists because of the way the two phases are defined. The four cases are as follow:

Case 1: No recombination happens ($g_{2}$ comes from the same chromosome as $g_{1}$).
  $$\left[\begin{array}{c} g_{1}\\ g_{2}\\ \vdots \\ g_{L} \end{array}\right]=\left[\begin{array}{c} G_{1,1,1}\\ G_{2,1,1}\\ \vdots \\ G_{L,m,s} \end{array}\right]$$

According to equation (12), when $i^{0}=i^{1}\;\text{and}\;m^{0}=m^{1}$, we have:

$$P\left(g_{l+1}=G_{l+1,m^{1},i^{1}}|g_{l}=G_{l,m^{0},i^{0}}\right)={\left(1-r_{l+1}\right)}^{2}\left(1-R_{l+1}\right)$$ (17)

$$\forall l\in \left\{1,\ldots ,L-1\right\},\;\forall i^{0},i^{1}\in \left\{1,2,\ldots ,S\right\},\;\forall m^{0},m^{1}\in \left\{1,2\right\}.$$

Using this definition equation (17) can be calculated as follow:

$$P\left(g_{l+1}=G_{l+1,m^{1},i^{1}}|g_{l}=G_{l,m^{0},i^{0}}\right)$$ (18)

$$=P\left(g_{l+1}=G_{l+1,m^{0},i^{0}}|g_{l}=G_{l,m^{0},i^{0}}\right)$$ (19)

$$=P\left(h_{l+1}=G_{l+1,m^{0},i^{0}},g_{l+1}=h_{l+1}|h_{l}=G_{l,m^{0},i^{0}},g_{l}=h_{l}\right)$$ (20)

$$=P\left(h_{l+1}=G_{l+1,m^{0},i^{0}}|h_{l}=G_{l,m^{0},i^{0}},g_{l}=h_{l}\right)\cdot P\left(g_{l+1}=h_{l+1}|h_{l}=G_{l,m^{0},i^{0}},g_{l}=h_{l}\right)$$ (21)

$$=P\left(h_{l+1}=G_{l+1,m^{0},i^{0}}|h_{l}=G_{l,m^{0},i^{0}}\right)P\left(g_{l+1}=h_{l+1}|g_{l}=h_{l}\right)$$ (22)

$$={\left(1-r_{l+1}\right)}^{2}\left(1-R_{l+1}\right)$$ (23)

Equation (19) comes from the fact that $i^{0}=i^{1}\;\text{and}\;m^{0}=m^{1}$. Equation (20) is derived from equation (19) because of the way *h* is defined. To find equation (21) from (20) independency is used. Finally, equation (22) is derived from (21) due to the fact that $h_{l+1}=G_{l+1,m^{0},i^{0}}$ is independent from $g_{l}=h_{l}$ and also $g_{l+1}=h_{l+1}$ is independent from $h_{l}=G_{l,m^{0},i^{0}}$.

Here, $R_{l}$ is the recombination frequency between allele *l* and allele l+1, ∀l∈{1,…,L−1} after (T−t)−2 number of generations and is calculated as:

$$R_{l}=1-P\left(g_{l+1}=h_{l+1}|g_{l}=h_{l}\right)$$ (24)

*Proof*.

$$R_{l}^{2}=0$$

$$R_{l}^{i}=1-\left(\left(1-R_{l}^{i-1}\right)\left(1-r_{l}\right)+\frac{r_{l}}{S/2}\right)\;\;\forall i\in \left\{3,4,\ldots ,\tau \right\}$$

Where $r_{l}$ is the $l^{th}$ recombination frequency for l∈{1,2,…,L−1} and *S* is number of breeding parents. From the above equations we obtain:

$$R_{l}=\frac{\left(S-2\right)\left(1-{\left(1-r_{l}\right)}^{T-t}\right)}{S}$$ (25)

This provides the proof for equation (13).

Case 2: Recombination happens within an individual ($g_{2}$ is coming from the other chromosome of the same individual where $g_{1}$ is coming from).
  $$\left[\begin{array}{c} g_{1}\\ g_{2}\\ \vdots \\ g_{L} \end{array}\right]=\left[\begin{array}{c} G_{1,1,1}\\ G_{2,2,1}\\ \vdots \\ G_{L,m,s} \end{array}\right]$$

According to equation (12), when $i^{0}=i^{1}\;\text{and}\;m^{0}=m^{1}$, we have:

$$P\left(g_{l+1}=G_{l+1,m^{1},i^{1}}|g_{l}=G_{l,m^{0},i^{0}}\right)=r_{l+1}\left(1-r_{l+1}\right)\left(1-R_{l+1}\right)$$ (26)

$$\forall l\in \left\{1,\ldots ,L-1\right\},\;\forall i^{0},i^{1}\in \left\{1,2,\ldots ,S\right\},\;\forall m^{0},m^{1}\in \left\{1,2\right\}.$$

Similarly, equation (26) can be calculated as follow:

$$P\left(g_{l+1}=G_{l+1,m^{1},i^{1}}|g_{l}=G_{l,m^{0},i^{0}}\right)$$ (27)

$$=P\left(h_{l+1}=G_{l+1,m^{1},i^{0}},g_{l+1}=h_{l+1}|h_{l}=G_{l,m^{0},i^{0}},g_{l}=h_{l}\right)$$ (28)

$$=P\left(h_{l+1}=G_{l+1,m^{1},i^{0}}|h_{l}=G_{l,m^{0},i^{0}}\right)P\left(g_{l+1}=h_{l+1}|g_{l}=h_{l}\right)$$ (29)

$$=r_{l+1}\left(1-r_{l+1}\right)\left(1-R_{l+1}\right)$$ (30)

Case 3: Recombination happens within the paired individual.
  $$\left[\begin{array}{c} g_{1}\\ g_{2}\\ \vdots \\ g_{L} \end{array}\right]=\left[\begin{array}{c} G_{1,1,1}\\ G_{2,1,2}\\ \vdots \\ G_{L,m,s} \end{array}\right],\text{or}\left[\begin{array}{c} g_{1}\\ g_{2}\\ \vdots \\ g_{L} \end{array}\right]=\left[\begin{array}{c} G_{1,1,1}\\ G_{2,2,2}\\ \vdots \\ G_{L,m,s} \end{array}\right]$$

According to equation (12), when $\frac{i^{0}}{2}=\frac{i^{1}}{2}$, we have:

$$P\left(g_{l+1}=G_{l+1,m^{1},i^{1}}|g_{l}=G_{l,m^{0},i^{0}}\right)=\frac{1}{2}\;r_{l+1}\left(1-R_{l+1}\right)$$ (31)

$$\forall l\in \left\{1,\ldots ,L-1\right\},\;\forall i^{0},i^{1}\in \left\{1,2,\ldots ,S\right\},\;\forall m^{0},m^{1}\in \left\{1,2\right\}.$$

Similarly, equation (31) can be calculated as follow:

$$P\left(g_{l+1}=G_{l+1,m^{1},i^{1}}|g_{l}=G_{l,m^{0},i^{0}}\right)$$ (32)

$$=P\left(h_{l+1}=G_{l+1,m^{1},i^{1}},g_{l+1}=h_{l+1}|h_{l}=G_{l,m^{0},i^{0}},g_{l}=h_{l}\right)$$ (33)

$$=P\left(h_{l+1}=G_{l+1,m^{1},i^{1}}|h_{l}=G_{l,m^{0},i^{0}}\right)P\left(g_{l+1}=h_{l+1}|g_{l}=h_{l}\right)$$ (34)

$$=\frac{1}{2}\;r_{l+1}\left(1-R_{l+1}\right)$$ (35)

Case 4: This case considers all possible remaining recombination.
  $$\left[\begin{array}{c} g_{1}\\ g_{2}\\ \vdots \\ g_{L} \end{array}\right]=\left[\begin{array}{c} G_{1,1,1}\\ G_{2,1,3}\\ \vdots \\ G_{L,m,s} \end{array}\right],\text{or}\left[\begin{array}{c} g_{1}\\ g_{2}\\ \vdots \\ g_{L} \end{array}\right]=\left[\begin{array}{c} G_{1,1,1}\\ G_{2,2,3}\\ \vdots \\ G_{L,m,s} \end{array}\right],\text{or}$$
  $$\left[\begin{array}{c} g_{1}\\ g_{2}\\ \vdots \\ g_{L} \end{array}\right]=\left[\begin{array}{c} G_{1,1,1}\\ G_{2,1,4}\\ \vdots \\ G_{L,m,s} \end{array}\right],\text{or}\left[\begin{array}{c} g_{1}\\ g_{2}\\ \vdots \\ g_{L} \end{array}\right]=\left[\begin{array}{c} G_{1,1,1}\\ G_{2,2,6}\\ \vdots \\ G_{L,m,s} \end{array}\right]$$

According to equation (12), when $\frac{i^{0}}{2}\neq \frac{i^{1}}{2}$, we have:

$$P\left(g_{l+1}=G_{l+1,m^{1},i^{1}}|g_{l}=G_{l,m^{0},i^{0}}\right)=\frac{R_{l+1}}{2\left(S-2\right)}$$ (36)

$$\forall l\in \left\{1,\ldots ,L-1\right\},\;\forall i^{0},i^{1}\in \left\{1,2,\ldots ,S\right\},\;\forall m^{0},m^{1}\in \left\{1,2\right\}.$$

Similarly, equation (36) can be calculated as follow:

$$P\left(g_{l+1}=G_{l+1,m^{1},i^{1}}|g_{l}=G_{l,m^{0},i^{0}}\right)$$ (37)

$$=P\left(h_{l+1}=G_{l+1,m^{1},i^{1}},g_{l+1}=h_{l+1}|h_{l}=G_{l,m^{0},i^{0}},g_{l}=h_{l}\right)$$ (38)

$$=P\left(h_{l+1}=G_{l+1,m^{1},i^{1}}|h_{l}=G_{l,m^{0},i^{0}}\right)P\left(g_{l+1}=h_{l+1}|g_{l}=h_{l}\right)$$ (39)

$$=\frac{1}{4}\times \frac{R_{l+1}}{\frac{S}{2}-1}$$ (40)

$$=\frac{R_{l+1}}{2\left(S-2\right)}$$ (41)
